# Oxygen desaturation during flexible bronchoscopy with propofol sedation is associated with sleep apnea: the PROSA-Study

**DOI:** 10.1186/s12931-020-01573-z

**Published:** 2020-11-19

**Authors:** Andrei M. Darie, Desiree M. Schumann, Marco Laures, Werner Strobel, Kathleen Jahn, Eric Pflimlin, Michael Tamm, Daiana Stolz

**Affiliations:** Clinic of Respiratory Medicine and Pulmonary Cell Research, University Hospital of Basel, Petersgraben 4, 4031 Basel, Switzerland

**Keywords:** Bronchoscopy, Sleep apnea, Tonometry, Hypoxia

## Abstract

**Background:**

Obstructive sleep apnea (OSA) is characterized by repetitive episodes of complete or partial obstruction of the upper airways during sleep. Conscious sedation for flexible bronchoscopy (FB) places patients in a sleep-like condition. We hypothesize that oxygen desaturation during flexible bronchoscopy may help to detect undiagnosed sleep apnea.

**Methods:**

Single-centre, investigator-initiated and driven study including consecutive patients undergoing FB for clinical indication. Patients completed the Epworth Sleepiness Scale (ESS), Lausanne NoSAS score, STOP-BANG questionnaire and the Berlin questionnaire and underwent polygraphy within 7 days of FB. FB was performed under conscious sedation with propofol. Oxygen desaturation during bronchoscopy was measured with continuous monitoring of peripheral oxygen saturation with ixTrend (ixellence GmbH, Germany).

**Results:**

145 patients were included in the study, 62% were male, and the average age was 65.8 ± 1.1 years. The vast majority of patients (n = 131, 90%) proved to fulfill OSA criteria based on polygraphy results: 52/131 patients (40%) had mild sleep apnea, 49/131 patients (37%) moderate sleep apnea and 30/131 patients (23%) severe sleep apnea. Patients with no oxygen desaturation had a significantly lower apnea–hypopnea index than patients with oxygen desaturation during bronchoscopy (AHI 11.94/h vs 21.02/h, p = 0.011). This association remained significant when adjusting for the duration of bronchoscopy and propofol dose (p = 0.023; 95% CI 1.382; 18.243) but did not hold when also adjusting for age and BMI.

**Conclusion:**

The severity of sleep apnea was associated to oxygen desaturation during flexible bronchoscopy under conscious sedation. Patients with oxygen desaturation during bronchoscopy might be considered for sleep apnea screening.

*Trial registration:* The Study was approved by the Ethics Committee northwest/central Switzerland, EKNZ (EK 16/13) and was carried out according to the Declaration of Helsinki and Good Clinical Practice guidelines. Due to its observational character, the study did not require registration at a clinical trial registry.

## Introduction

Obstructive sleep apnea (OSA) is a condition caused by repetitive episodes of partial or complete airway collapse during sleep. The prevalence rates of OSA have increased substantially over the last decades, ranging from 14 to 55% depending on the age and gender of the patient population [1]. The significant relationship with cardiovascular and metabolic diseases forms the major health burden associated with OSA which leads to substantial morbidity and mortality [2–4].

Symptoms of OSA include increased daytime sleepiness, fatigue, irritability, inattention and a decrease in cognitive function resulting in a highly heterogeneous disease with multiple phenotypes [5–7]. Although OSA is a common problem, the fact that the respiratory events, (apnea and hypopnea) occur during sleep, results in an unawareness of- and underdiagnosed disease [8]. Various questionnaires including the Epworth Sleepiness scale (ESS), [9]; Berlin questionnaire [10]; the snoring, tiredness, observed apnea, high blood pressure, body mass index (BMI), age, neck circumference, and male gender (STOP-BANG) questionnaire [11] and scores Lausanne NoSAS score [12] and the Multivariable Apnea Prediction (MVAP) score [13] exist to aid in identifying patients with OSA.

Flexible bronchoscopy (FB) is a generally safe minimally invasive procedure used to assess, diagnose and treat patients with respiratory disease [14]. Transient hypoxemia due to upper airway obstruction is known to occur in patients undergoing flexible bronchoscopy [15–18]. However, the association between transient hypoxemia during FB and the presence of sleep apnea remains unexplored.

We hypothesize that transient hypoxemia during flexible bronchoscopy under conscious sedation might be associated with the apnea–hypopnea index (AHI) assessed by polygraphy and therefore could inform about the presence of sleep apnea.

## Methods

### Patient selection

This was a prospective, investigator-initiated and driven, single-centre cross-sectional study performed at the Clinic of Respiratory Medicine and Pulmonary Cell Research at the University Hospital of Basel between October 2018 and August 2019. Patients older than 18 years undergoing a diagnostic flexible bronchoscopy were sequentially recruited. Exclusion criteria were hypoxemia at rest defined as an oxygen saturation of < 90% in room air, rapid fatal disease, any disease or condition precluding the initiation of continuous positive airway pressure (CPAP) therapy within the next 6 months and a new onset of cardio-respiratory symptoms (“unstable state”) as defined by a deterioration of cardio-vital signs within the last 48 h. Patients who required intubation during the bronchoscopy were withdrawn from the study.

The Study was approved by the Ethics Committee northwest/central Switzerland, EKNZ (EK 16/13) and was carried out according to the Declaration of Helsinki and Good Clinical Practice guidelines. Due to its observational character, the study did not require registration at a clinical trial registry.

Collected data included patient demographic data, current pulmonary function test, arterial blood gas analysis, oxygen saturation during bronchoscopy and all information related to the bronchoscopy. A targeted physical examination was performed on all subjects including BMI, neck circumference and examination of the oral cavity to record the oropharyngeal Mallampati score. All patients completed the ESS, and Berlin Questionnaire. The NoSAS Score and STOP-BANG score were calculated from measurements and data collected from the patient. The ESS is a self-administered questionnaire that provides insight into the subject`s general level of daytime sleepiness. [9] The ESS is applied by rating the probability of falling asleep during eight different scenarios usually encountered in daily life. A meta-analysis found the sensitivity of the ESS questionnaire ranges between 47 and 52% depending on the severity of OSA [19]. The Berlin Questionnaire is a validated instrument for screening patients at risk for sleep apnea. In this questionnaire, snoring and observed apnea (first domain, five questions), daytime sleepiness (second domain, four questions) and BMI and hypertension (third domain, one question and information on height and weight) are assessed. The Berlin Questionnaire is positive when two of the three domains are positive. It has a sensitivity of 86% in predicting patients with a respiratory disturbance index (RDI) above 5/h [10]. The NoSAS score is a screening tool used to identify persons at risk for sleep apnea [12]. It uses information about neck circumference, BMI, snoring, age and gender to predict sleep apnea. A score of 8 points or higher is predictive of sleep apnea. The NoSAS score has a sensitivity of 79–85% [12]. The STOP-Bang questionnaire is a concise screening tool for OSA. It is self-reportable and includes four subjective features (Snoring, Tiredness, Observed apnea and presence of high blood pressure) and four demographic queries (BMI, Age, Neck circumference and Gender). Every item can be scored with zero or one point with a maximum score of eight points. Using a cutoff of ≥ 3, the STOP-BANG score has a sensitivity of 84% in detecting OSA [11].

### Sleep apnea evaluation

Polygraphy, was used to screen the patients for sleep apnea (WatchPAT™200 Unified, Itamar medical) and was performed in an in-patient setting within 7 days before the bronchoscopy. The following parameters were measured via three points of contact: peripheral arterial tonometry signal, heart rate, oximetry, actigraphy, body position, snoring and chest motion. AHI, RDI and oxygen desaturation index (ODI) based on true sleep time and sleep staging were assessed. Mild sleep apnea was defined as 5 ≤ AHI ≤ 15, moderate sleep apnea as 15 < AHI ≤ 30 and severe sleep apnea as AHI > 30. Sleep apnea syndrome is defined as AHI ≥ 5/h + symptoms of daytime sleepiness [8]. We calculated sleep apnea syndrome using the ESS, Berlin score, NoSAS score and STOP-BANG score.

### Bronchoscopy

All patients were assessed prior to the bronchoscopy by a physician or a member of the nursing team trained in anesthesiology and graded according to the American Society of Anesthesiologists (ASA) criteria. The bronchoscopy was performed trans-nasally or trans-orally with the patients in a semi-recumbent position. Pulse oximetry and electrocardiography were recorded continuously and blood pressure was monitored automatically and non-invasively every 5 min. These readings were recorded with the patient monitor, ixTrend Express (ixitos GmbH, Berlin Germany). Supplemental oxygen at 4 l/min was supplied through a nasal cannula to all patients, and oxygen delivery was increased gradually up to 12 L/min when oxygen saturation dropped below 90%. Lidocaine 2% gel was applied as a nasal anesthesia. The bronchoscopist introduced 5-ml aliquots of 1% lidocaine over the vocal cords, the trachea and both left and right main bronchi. In certain patients, up to 4 mg hydrocodone was administered prior to the procedure [20]. Nurses trained in endoscopy performed the conscious sedation with propofol, which was applied intravenously through an infusion-pump and in addition, propofol-boluses were applied and titrated to achieve adequate sedation (onset of ptosis) for the bronchoscopy. The amount of propofol applied was further titrated during the bronchoscopy in order to maintain conscious sedation, defined as a decreased state of consciousness that minimizes discomfort [21–25]. Hydrocodone was usually applied as a cough-suppressant. [26, 27]

The event of a relevant oxygen desaturation during conscious sedation for flexible bronchoscopy was characterized as a minimal transcutaneous oxygen saturation < 90% for ≥ 5 s. For additional explorative analyses we also analysed oxygen desaturations < 90% for ≥ 1 min, desaturations ≤ 88% for 5 s and a ≥ 4% decrease in oxygen saturation from baseline. During the bronchoscopy the following additional parameters were noted: audible snoring for ≥ 10 s, non-invasive ventilation, chin support and witnessed apnea longer than 10 s.

Bronchoscopy complications were defined as bleeding requiring intervention, need for non-invasive ventilation, need to abort the examination, transfer to the intensive care unit, pneumothorax or death. The decision to discontinue the bronchoscopy, to provide chin support or non-invasive ventilation was taken by the bronchoscopist. [24, 27]

### Statistical analysis

Differences in dichotomous variables were evaluated using the Chi-squared test or Fisher exact test, as appropriate. Normally distributed parameters were analyzed using the Student t-test for equality of means. All other continuously non-normally distributed parameters were evaluated using the non-parametric Mann–Whitney U test or Kruskal–Wallis test, as appropriate. The association between oxygen desaturation and a diagnosis of sleep apnea was evaluated by linear regression using a univariate model and a multivariate model adjusting for the duration of the bronchoscopy and amount of propofol used. The Statistical Package for Social Sciences Program (SPSS Inc, version 22 for Windows) was used. All tests are two-tailed, a P-value < 0.05 was considered significant. Results are expressed as mean (SEM) or median (interquartile range) unless stated otherwise.

### Sample size calculation

We assumed that patients without desaturation below 90% during flexible bronchoscopy under conscious sedation would depict an AHI of 5/h ± 18 during polygraphy whereas patients with oxygen desaturation below 90% during FB would depict an AHI of 15/h ± 18. We considered an uneven distribution of desaturation during bronchoscopy of 70–30%. A total of 131 patients (77 + 54) would need to be included in the study to achieve a level of significance of 5% and a power of 80%. Considering a lost-to-follow-up of 5%, a total of 145 patients (85 + 60) was planned for inclusion in the study.

## Results

### Baseline characteristics

**Fig. 1:**
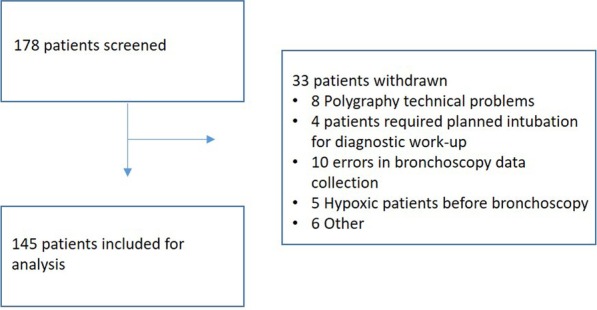
A schematic diagram according to the CONSORT recommendations depicting patient inclusion in the study

The number of patients screened for the study totaled 178. Of these 178 patients, 33 patients were excluded and 145 patients were included in the study (Fig. 1). The included population consisted of 90 (62%) males, the mean BMI was 25.6 ± 0.4 kg/m^2^ and a third of the patients were current smokers (n = 48; Table 1). The majority of the patients had an ASA-Score III. A Mallampati score of 3–4 was present in 105/145 (72%) of the patients. The total number of patients with observed snoring during the bronchoscopy (n = 107; 74%) was higher than the number of patients with self-reported snoring (n = 74; 51%) and lower than the number of patients recorded snoring using polygraphy (n = 121; 83%). The most prevalent comorbidity was chronic obstructive pulmonary disease (COPD) followed by renal disease and coronary artery disease (Table 1).

**Table 1:** Basic characteristics of the patients included in the study

	N(%), mean ± SEM
Age, years	65.8 ± 1.1
Male	90 (62)
*Smoking status*	
Current smoker	48 (33)
Past smoker	64 (44)
Never smoker	33 (23)
*ASA class*	
I–II	38 (26)
III–V	107 (74)
BMI	25.6 ± 0.4
*Mallampati score*	
1–2	40 (27.6)
3–4	105 (72.4)
Neck circumference, cm	39.7 ± 0.37
*Snore*	
According to patient	74 (51)
According to sleep report	121 (83)
*Comorbidities*	
Alcoholism	18 (12)
Cerebral vascular disease	18 (12)
Chronic obstructive pulmonary disease	71 (49)
Congestive heart failure	11 (8)
Coronary artery disease	27 (19)
Depression	21 (14)
Diabetes mellitus	21 (14)
Liver disease	20 (14)
Malignant solid tumour	7 (5)
Pulmonary neoplasia	3 (2)
Renal disease	37 (26)
Rheumatological disease	10 (7)
Other*	16 (11)

*Other includes drug abuse, hematologic malignancy, HIV and immunosuppression

Twenty-eight patients (19%) had an elevated ESS whereas the STOP-BANG was suggestive of 93/145 (64%) high-risk patients (Table 2). Nineteen/28 (68%), 18/28 (64%) and 22/28 (79%) patients with an elevated ESS were concomitantly ranked as high-risk by the Berlin score, the Lausanne No-SAS and the STOP-BANG score respectively.

**Table 2:** Data regarding the flexible bronchoscopy and questionnaire scores

	N(%)
*Bronchoscopy*	
Chin support	116 (80)
Non-invasive ventilation	4 (3)
Witnessed apnea	7 (5)
Complications	7 (5)
*Procedures*	
Bronchoalveolar lavage	135 (93)
Bronchial washing	15 (10)
Endobronchial biopsy	60 (41)
Transbronchial lung biopsy	32 (22)
Endobronchial ultrasound needle aspiration	54 (37)
Bronchial brushing	31 (21)
Transbronchial needle aspiration	14 (10)
Endoscopic lung volume reduction	5 (3)
Bronchial thermoplasty	2 (1)
Radial probe endobronchial ultrasound	6 (4)
*Questionnaires and Scores*	
Epworth score ≥ 10	28 (19)
Berlin score – high risk	85 (59)
NoSAS ≥ 9	81 (56)
STOP-BANG – high risk	93 (64)

The mean recorded time during the polygraphy was 7.2 ± 0.12 h with an average sleep time of 5.3 ± 0.12 h. The snore volume was on average 44 ± 0.76 dB and the average AHI was 20.3 ± 1.2. Information regarding sleep parameters can be found in Additional file 1: Table 1.

**Fig. 2:**
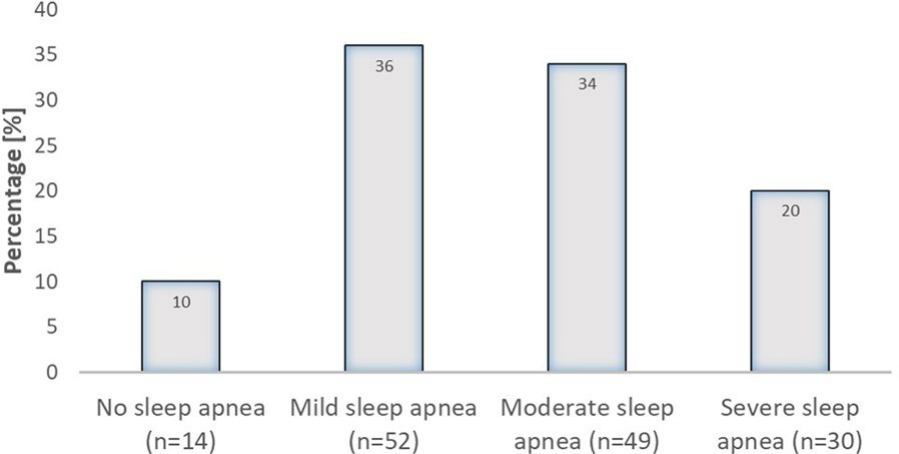
Incidence of sleep apnea in our cohort. Mild sleep apnea was defined as 5/h < AHI ≤ 15/h; moderate sleep apnea was defined as 15/h < AHI ≤ 30/h and severe sleep apnea was defined as AHI > 30/h

A normal AHI was measured in 14/145 (10%) of the patients. We detected mild sleep apnea in 52/145 patients (36%), moderate sleep apnea was observed in 49/145 patients (34%) and severe sleep apnea was seen in 30/145 patients (20%; Fig. 2).

There was a significant association between gender and incidence of sleep apnea (p = 0.034) with a higher incidence in males (94%) than females (84%). Oxygen desaturation during bronchoscopy, however, was not associated with gender (p = 0.56). When adjusting for Mallampati score and duration of bronchoscopy, oxygen desaturation during the bronchoscopy still had a significant association with AHI (p = 0.035). Logistic regression analyzing Mallampati score and its association with sleep apnea or no sleep apnea, showed no association whether as a discrete, categorical variable (p = 0.832) or when grouped according to severity (p = 0.477).

There was no association between the prevalence of sleep apnea and snoring as measured during the polygraphy (Pearson Chi-square, p = 0.729). Conversely, there was a significant correlation between AHI and age (Spearman Rho correlation coefficient = 0.254; p = 0.002), BMI (Rho = 0.347; p < 0.001), forced vital capacity (FVC) % predicted (Rho = − 0.188; p = 0.023); and total lung capacity (TLC) %predicted (Rho = − 0.277; p < 0.001). There was no correlation between AHI and pre or post bronchodilator forced expiratory volume in 1 s (FEV_1_).

Bronchoscopy was performed on average 1.5 ± 0.08 days after polygraphy and had a mean duration of 23.5 ± 1.3 min. The average dosage of propofol needed for sedation was 323 ± 16.5 mg and 105/145 patients (72%) also received hydrocodone as a cough suppressant. There was no association (p = 0.35) between the administration of hydrocodone and sleep apnea. Of the patients receiving hydrocodone, 93/105 (89%) had sleep apnea. Of the patients not administered hydrocodone, 38/40 (95%) had sleep apnea. Hydrocodone had no effect on the incidence of oxygen desaturations (Pearson Chi-square, p = 0.75). Chin support was performed on 116/145 patients (80%), and 4/145 patients (3%) required non-invasive ventilation.

**Fig. 3:**
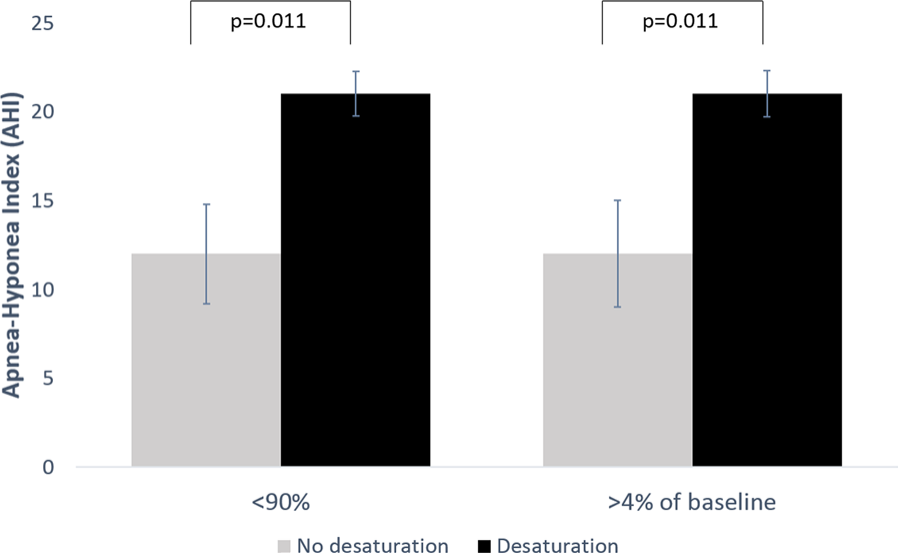
Apnea–Hypopnea Index (AHI) was significantly higher in patients who had any SaO_2_ < 90% and in patients with a drop in SaO_2_ of ≥ 4% from baseline compared to patients who did not develop hypoxemia

During bronchoscopy, an oxygen saturation of < 90% for ≥ 5 s was measured in 132/145 patients (91%; Fig. 3), an oxygen saturation < 88% for ≥ 5 s was measured in 123/145 patients (85%) and a decrease in oxygen saturation of ≥ 4% of the baseline oxygen saturation was measured in 132/145 patients (91%).

There was a significant difference in age between the patients who remained normoxemic during the bronchoscopy and the patients who had an oxygen saturation < 90% (57.9 ± 3.5 vs. 66.6 ± 1.1 years; p = 0.017), < 88% (56.0 ± 3.1 vs. 67.5 ± 1.1 years; p = 0.001) and ≥ 4% decrease from baseline (57.9 ± 3.5 vs. 66.6 ± 1.2 years; p = 0.017). There was also a difference in BMI between the patients who remained normoxemic and the patients who had an oxygen saturation < 90% (23.5 ± 1.3 vs 25.8 ± 0.42 kg/m^2^; p = 0.055), and those who had a ≥ 4% decrease from baseline (23.5 ± 1.3 vs. 25.8 ± 0.4 kg/m^2^; p = 0.055). Remarkably, lung function parameters such as post-bronchodilator FEV_1_ (2.1 ± 0.3 vs. 2.3 ± 0.5 L; p = 0.248), post-bronchodilator FVC (3.4 ± 0.4 vs. 3.0 ± 0.1 L; p = 0.262), post-bronchodilator TLC (100 ± 8.5 vs. 107 ± 2.0%; p = 0.355) and post-bronchodilator vital capacity (VC) (3.6 ± 0.35 vs. 3.3 ± 0.1 L; p = 0.408) did not differ significantly between the normoxemic and the hypoxemic patients. Patients with oxygen desaturation during the bronchoscopy had significantly less sleep time during the polygraphy (Table 3).

**Table 3:** Baseline characteristics stratified according to whether patients depicted at least one desaturation during bronchoscopy

	No desaturation n = 13	Desaturation n = 132	p Value
Sleep time during polygraphy (h)	6.0 ± 0.3	5.2 ± 0.1	**0.045**
*Snore*			
According to patient	3 (23)	71 (54)	**0.035**
According to sleep report	8 (62)	113 (86)	0.790
Time between polygraphy/bronchoscopy (days)	1.5 ± 0.2	1.5 ± 0.08	0.775
*Bronchoscopy procedures*			
Bronchoalveolar lavage	15 (11)	120 (89)	0.266
Bronchial washing	1 (7)	14 (93)	0.621
Endobronchial biopsy	8 (13)	52 (87)	0.321
Transbronchial lung biopsy	1 (3)	31 (97)	0.129
Endobronchial ultrasound needle aspiration	3 (6)	51 (94)	0.145
Bronchial brushing	2 (6)	29 (94)	0.422
Transbronchial needle aspiration	1 (7)	13 (93)	0.679
Endoscopic lung volume reduction	0 (0)	5 (100)	0.440
Bronchial thermoplasty	0 (0)	2 (100)	0.629
Radial probe endobroncial ultrasound	0 (0)	6 (100)	0.395
Bronchoscopy duration (min)	11.05 ± 1.99	24.7 ± 81.37	**0.001**
Total Propofol dose (mg)	210.9 ± 31.4	334 ± 17.6	**0.019**
*Questionnaires and Scores*			
Epworth score ≥ 10	4 (31)	24 (18)	0.273
Berlin score – high risk	6 (46)	79 (60)	0.339
NoSAS ≥ 9	4 (31)	77 (58)	0.056
STOP-BANG-high risk**	5 (38)	88 (67)	**0.043**

P-values < 0.05 are shown in bold

**STOP-BANG snoring, tired, observed, pressure, body mass index, age, neck size, gender

Patients with oxygen desaturation < 90% had significantly higher AHI values compared to patients with no oxygen desaturation (Fig. 3). Thus, there was a significant association between AHI and oxygen desaturation < 90% (β 9.082 CI 0.982–17.182, p = 0.028) suggesting that patients presenting oxygen desaturation < 90% during bronchoscopy had an AHI 9.1/h higher than patients with no oxygen desaturation during bronchoscopy. This association remained significant after adjusting for the duration of the procedure and for the administered propofol dose (β 9.813 CI 1.382–18.243 p = 0.023) and also held true when examining desaturation < 90% for ≥ 1 min (Additional file 1: Fig. 1). The association disappeared when adjusting for gender, age and BMI together (β 5.729 CI − 2.261–13.719; p = 0.159).

When using polygraphy as a reference standard, oxygen desaturation during bronchoscopy had a sensitivity of 92% and a specificity of 14% to diagnose sleep apnea. The average score for the Epworth sleepiness questionnaire and the NoSAS score according to oxygen desaturation or no oxygen desaturation is depicted in Additional file 1: Table 2. STOP-BANG had the highest sensitivity and ESS the lowest sensitivity to diagnose sleep apnea when using either AHI or oxygen desaturation during bronchoscopy as reference standards (Table 4).

**Table 4:** Sensitivity, specificity, predictive values and likelihood ratios of the various questionnaires in relation to AHI and desaturation during bronchoscopy

	Berlin Questionnaire	Epworth Sleepiness Scale	Lausanne NoSAS	STOP-BANG
Using sleep apnea assessed by polygraphy as reference standard				
Sensitivity	59	20	58	69
Specificity	43	86	64	79
Positive predictive value	91	93	94	97
Negative predictive value	10	10	14	21
Positive likelihood ratio	1.0	1.6	1.6	3.2
Negative likelihood ratio	0.96	0.94	0.66	0.4
Using desaturation during bronchoscopy as reference standard				
Sensitivity	60	18	58	67
Specificity	54	69	69	62
Positive predictive value	93	86	95	95
Negative predictive value	12	7.7	14	15
Positive likelihood ratio	1.3	0.59	1.9	1.8
Negative likelihood ratio	0.74	1.2	0.61	0.54

The sensitivity and specificity of oxygen desaturation < 90% during bronchoscopy to diagnose obstructive sleep apnea syndrome (OSAS) using AHI > 5/h and a positive symptom score as a reference standard is depicted in Table 5.

**Table 5:** The sensitivity and specificity of desaturation during bronchoscopy for determining OSAS using AHI > 5/h and a positive symptom score using the Berlin questionnaire, Epworth sleepiness scale, Lausanne NoSAS and STOP-BANG as a reference standard

	OSAS using Berlin Questionnaire	OSAS using Epworth Sleepiness Scale	OSAS using Lausanne NoSAS	OSAS using STOP-BANG
Sensitivity	92	85	95	94
Specificity	10	7.6	13	15
Positive predictive value	54	17	55	64
Negative predictive value	54	69	69	62
Positive likelihood ratio	1.0	0.9	1.1	1.1
Negative likelihood ratio	0.8	1.97	0.38	0.40

The values changed when taking into consideration sleep apnea syndrome with a pAHI ≥ 15 (Additional file 1: Table 3).

## Discussion

Transient hypoxemia is known to occur in patients undergoing FB, however, the association between transient hypoxemia during FB and OSA is unknown. We found that patients who experienced hypoxemia during FB under conscious sedation had increased AHI in the polygraphy. The association between oxygen desaturation < 90% during bronchoscopy and AHI remained significant after adjustment for duration of the procedure and propofol dose. Accordingly, patients presenting oxygen desaturation < 90% during bronchoscopy had a 9.1/h higher AHI than those not presenting oxygen desaturation during the procedure.

Hypoxia is the most commonly cited adverse event in patients undergoing bronchoscopy [16, 17, 28]. The occurrence of hypoxemia was highly prevalent during FB in our study, with an oxygen saturation of < 90% for ≥ 5 s measured in 132 patients (91%). This reflects the severity and multi-morbidity of the patient population included in the study with almost 50% of the participants suffering from COPD and 74% classified as ASA Class III or higher. In a recent study by Cho et al. [18] they had an incidence of hypoxemia of 35% during moderate sedation bronchoscopy. Their population consisted of 35% ASA III patients and 65% ASA II patients. Although ASA classification was introduced as a subjectively determined marker of general health used to evaluate perioperative morbidity [29], it was also reported as a predictor for occurrence of adverse events, including hypoxemia, during endoscopic procedures [30–32]. Nonetheless, the occurrence of hypoxemia during flexible bronchoscopy with sedation is similar in studies with and without supplemental oxygen [16, 33–35]. A recent study in Norway has shown that when patients chose to have no sedation, it significantly increased unplanned interventions during the bronchoscopy [17].

Although the safety and efficacy of propofol used as a sedative during bronchoscopy has been explored [21–23, 25], airway collapsibility is affected by propofol in a dose dependent manner making proper titration a decisive factor in avoiding overestimating the severity of airway obstruction and implicitly OSA [36]. The mean dose of propofol required to achieve proper sedation in the present study was slightly higher than previously reported [21, 22, 37]. However, the number of complex procedures performed during bronchoscopy has increased compared to earlier studies, thus underpinning a higher sedative requirement. In addition the intravenous continuous infusion of propofol, which is as safe as bolus administration, is known to be associated with an increased dosage during FB [25]. Endoscopic procedures known as drug-induced sleep endoscopy (DISE) are widely recognized as diagnostic instruments for OSA [38] due to induction of airway obstruction and collapse during sedation [39]. Propofol is the recommended pharmacologic agent for DISE [38]. The use of propofol to induce a sleep-like state, mimics the drop in oxygen saturation seen during polysomnography and the respiratory events are comparable to the respiratory events seen during polysomnography [40, 41].

We found that the association between oxygen desaturation during bronchoscopy and higher AHI remained significant after adjusting for propofol dose even though patients do not enter REM sleep during the bronchoscopy. It has been shown that most of the respiratory events in sleep apnea occur during N2 sleep [40, 42–44].

Sleep apnea assessed using polygraphy, was highly prevalent in our population with 90% of patients having an AHI above 5/h, even though BMI and neck circumference were normal. Similar results were found by Cho et al. [18] who, using the STOP-BANG score ≥ 3 found that 67% of their patients were at high risk of sleep apnea even though the average neck circumference and BMI were in the normal range. The high prevalence of sleep apnea found in the present study is similar to values previously reported [45]. Although the subjects enrolled in the other study had similar BMI (25.6 vs 25.6) and neck circumference (36.9 vs 39.7) the patients in our study were older (57 vs 66 years) [45]. In addition, a moderate to severe sleep apnea was observed in 54% of our patients and a gender difference in prevalence was evident. These results are similar to previously published rates of sleep apnea in the general population [45–47]. The difference in sleep apnea incidence between males and females may be due to differences in upper airway mechanical or neuromuscular properties, chemoreflex control of breathing or sex hormone levels [46]. There was, however, no difference in the occurrence of oxygen desaturation during bronchoscopy when comparing males and females.

The Mallampati index is a quick instrument for assessing airway patency before intubation [48]. There is growing data pointing out the association of a higher Mallampati index to severity of OSA [49, 50]. Most of the patients enrolled in our study (72%) had a Mallampati index of three or more. In the study by Wang et al., they found that 85% of patients with sleep apnea had a Mallampati score of three or higher [51]. In addition, Sharara et al. [52] also found a Mallampati score of at least three with a prevalence of 83% in a population of snorers. Furthermore, in our study the Mallampati score was assessed in the supine position, which may have led to an increased number of patients being graded with a Mallampati score of three or four. In a study by Bindra et al. [53] the number of patients with a Mallampati score of three or four, more than doubled when assessed in the supine position. We, however, found no association between Mallampati index and sleep apnea or no sleep apnea.

The incidence of OSAS ranged between 15 and 59% depending on the questionnaire or score used. This was much higher than the incidence reported in the literature [54–56] and could be due to the population studied, as our population was older and had more severe disease.

In our study, patients who had hypoxemia during flexible bronchoscopy under conscious sedation had increased AHI as measured by polygraphy. Harvin et al. found that patients with a high risk of sleep apnea as assessed by the Berlin score had a higher rate of hypoxemia during conscious sedation for colonoscopy [28]. It is unclear whether this association remained true after adjusting for confounding variables. Cho et al. found an association between a high risk for sleep apnea, assessed using the STOP-BANG score, and cardiopulmonary events, such as hypoxia, during bronchoscopy [18]. As with male gender, there is also a known association between sleep apnea, age and BMI [47]. Thus both characteristics are also included in sleep apnea screening tools like Lausanne NoSAS [12] and the STOP-Bang questionnaire [11]. The association between AHI and oxygen desaturation disappeared in a multivariate analysis when adjusting for age and BMI and duration of bronchoscopy. Whereas age might be a confounder, BMI is probably related to effect modification, as risks tend to increase as a certain cut-off is reached.

In our non-selected group of patients, we had an incidence of overlap syndrome (COPD + sleep apnea) in 66/145 patients (46%). This is similar to incidence rates observed by Wang et al. [57] and Zhang et al. [58].

In our study, the Berlin Questionnaire had a 59% sensitivity and 43% specificity to identify sleep apnea as assessed by polygraphy. Only the STOP-BANG score was associated with oxygen desaturation during flexible bronchoscopy. This corroborates the findings of Cho et al. [18]. The sensitivity and specificity of ESS was low and no association was seen with AHI. Conversely, Johns MW [9] found that a higher ESS score correlates with the respiratory index measured during polysomnography and with the minimum oxygen saturation measured during the night.

The key message of the present study is that oxygen desaturation during endoscopic procedures, in particular flexible bronchoscopy, should prompt the treating physician to screen for sleep apnea. This could prove to be essential especially if we consider the prevalence of sleep apnea in this study, which is higher than in other epidemiologic studies. It also underlines that foremost in patient populations with high number of comorbidities, some of the screening tools, as for example the questionnaires evaluated in our study (NoSAS, STOP-BANG and ESS), could underestimate the prevalence of sleep-disordered breathing.

Limitations of this study include the fact that the sedation was applied by a trained nurse on the basis of clinical evaluation of sedation level (i.e. to achieve ptosis). This may result in an excessive sedation. Also, this was a single-center study in a tertiary hospital with patients with high ASA scores, therefore, the results may not be applicable to the general population.

Sleep apnea was determined by polygraphy and not polysomnography. However, polygraphy is confirmed as a viable diagnostic tool for sleep apnea and is included in the clinical algorithm for implementation of clinical practice guidelines by the American Academy of Sleep Medicine [59]. The high agreement ratio between polygraphy and polysomnography [60] may be attributable to the development of the peripheral arterial tonometry signal (PAT) which addresses the inability of other home sleep apnea tests to record and stage sleep. The algorithm used for sleep/wake detection and categorizing sleep stages REM/NREM have been described [60, 61]. The overall agreement in detecting REM/NREM sleep was 88.7%/88.6%, respectively when compared to polysomnography [62]. The limitation in using WatchPAT is that it is unable to reliably differentiate sleep apnea into obstructive, central or obstructive/central.

Another possible limitation is that since the exclusion criteria were few and the possibility to participate in the study was open, prevalently patients concerned about having a sleep disorder would participate. We don’t feel that this bias existed in our population. According to the Epworth Sleepiness Scale results, which is the reference standard used to determine the symptoms experienced by the patient, only 28/145 patients in the study actually had symptoms of a sleep disorder. Most of the patients had no symptoms even though they were at high risk of developing sleep apnea as shown with the STOP-BANG score, in which 93/145 (64%) patients had a score ≥ 3. In addition, 5.6% of the screened patients refused participation.

The strength of our study is the objective assessment of sleep apnea before undergoing bronchoscopy and the clinical applicability of study results.

In conclusion, oxygen desaturations during FB are associated with more severe sleep apnea. This association remains significant after adjusting for sedative dose and duration of procedure. It appears justifiable to consider sleep apnea screening for patients with oxygen desaturation during bronchoscopy.

## Supplementary information

**Additional file 1: Table 1.** Variables related to sleep apnea measured during REM sleep and non-REM sleep.** Table 2.** Epworth sleepiness score and Lausanne NoSAS were not associated with oxygen desaturation during conscious bronchoscopy. **Table 3.** The sensitivity and specificity of OSAS as calculated from AHI > 15/h and a positive symptom score using the Berlin questionnaire, Epworth sleepiness scale, Lausanne NoSAS and STOP-BANG and using desaturation as a reference standard. **Figure 1.** Apnea-Hypopnea Index (AHI) was significantly higher in patients who had any SaO2 < 90% for ≥ 1min compared to patients who did not develop hypoxemia.

## Data Availability

The datasets used and/or analysed during the current study are available from the corresponding author on reasonable request.

## Abbreviations

AHI: Apnea–hypopnea index
ASA: American Society of Anesthesiologists
BMI: Body mass index
COPD: Chronic obstructive pulmonary disease
CPAP: Continuous positive airway pressure
DISE: Drug-induced sleep endoscopy
ESS: Epworth sleepiness scale
FB: Flexible bronchoscopy
FEV1: Forced expiratory volume in 1 second
FVC: Forced vital capacity
OSA: Obstructive sleep apnea
OSAS: Obstructive sleep apnea syndrome
STOP-BANG: Snoring, tired, observed, pressure, body mass index, age, neck circumference, gender
