# Supplementary material for: Oxygen desaturation during flexible bronchoscopy with propofol sedation is associated with sleep apnea: the PROSA-Study
Source: Respir Res. 2020 Nov 19;21:306. doi: 10.1186/s12931-020-01573-z (PMC7678046; doi:10.1186/s12931-020-01573-z)
Supplement: Supplementary file 1 — Additional file 1: Table 1. Variables related to sleep apnea measured during REM sleep and non-REM sleep. Table 2. Epworth sleepiness score and Lausanne NoSAS were not associated with oxygen desaturation during conscious bronchoscopy. Table 3. The sensitivity and specificity of OSAS as calculated from AHI > 15/h and a positive symptom score using the Berlin questionnaire, Epworth sleepiness scale, Lausanne NoSAS and STOP-BANG and using desaturation as a reference standard. Figure 1. Apnea-Hypopnea Index (AHI) was significantly higher in patients who had any SaO2 < 90% for ≥ 1min compared to patients who did not develop hypoxemia. [file 12931_2020_1573_MOESM1_ESM.docx]

Additional:

Figure 1: Apnea-Hypopnea Index (AHI) was significantly higher in patients who had any SaO2 <90% for ≥1min compared to patients who did not develop hypoxemia
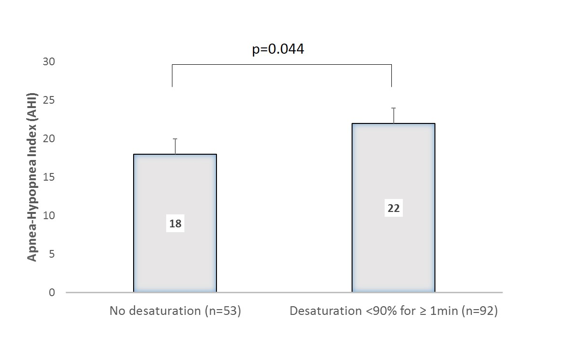


Table 1: Variables related to sleep apnea measured during REM sleep and non-REM sleep .

|  | Mean±SEM |
| --- | --- |
| pRDI_REM | 32.2±1.7 |
| pRDI_NREM | 20.0±1.4 |
| pRDI_Total | 22.8±1.2 |
| pAHI_REM | 29.1±1.8 |
| pAHI_NREM | 17.7±1.3 |
| pAHI_Total | 20.3±1.2 |
| ODI_REM | 15.1±1.4 |
| ODI_NREM | 7.6±0.86 |
| ODI_Total | 9.7±0.84 |

RDI – respiratory disturbance index; REM – rapid eye movement; NREM – non-rapid eye movement; AHI – apnea-hypopnea index; ODI – oxygen desaturation index

Table 2: Epworth sleepiness score and Lausanne NoSAS were not associated with oxygen desaturation during conscious bronchoscopy

|  | Epworth sleepiness score  Median (IQR) | p-value | Lausanne NoSAS  Median (IQR) | p-value |
| --- | --- | --- | --- | --- |
| Desaturation ≤90% | 5.0 (3.0; 8.0) |  | 9.5 (6.0; 13.0) |  |
| No desaturation ≤90% | 6.0 (5.0; 11.0) | 0.082 | 6.0 (5.0; 11.0) | 0.142 |
| Desaturation ≤88% | 5.0 (3.0; 8.0) |  | 9.0 (6.0; 13.0) |  |
| No desaturation ≤88% | 6.0 (4.0; 10.25) | 0.061 | 7.5 (4.0; 11.0) | 0.157 |
| Desaturation ≥4% of baseline | 5.0 (3.0; 8.0) |  | 9.45±0.40 |  |
| No desaturation ≥4% of baseline | 6.0 (5.0; 11.0) | 0.082 | 7.5±1.14 | 0.142 |

**Table 3**: The sensitivity and specificity of OSAS as calculated from AHI > 15/h and a positive symptom score using the Berlin questionnaire, Epworth sleepiness scale, Lausanne NoSAS and STOP-BANG and using desaturation as a reference standard.

|  | **OSAS using Berlin Questionnaire** | **OSAS using Epworth Sleepiness Scale** | **OSAS using Lausanne NoSAS** | **OSAS using STOP-BANG** |
| --- | --- | --- | --- | --- |
| Sensitivity | 96 | 94 | 98 | 97 |
| Specificity | 12 | 9.4 | 13 | 13 |
| Positive Predictive Value | 36 | 13 | 41 | 44 |
| Negative Predictive Value | 85 | 92 | 92 | 85 |
| Positive Likelihood ratio | 1.1 | 1.0 | 1.9 | 1.1 |
| Negative Likelihood ratio | 0.33 | 0.64 | 0.61 | 0.23 |
